# Informing decisions on the purchase of equipment used by health services in response to incidents involving hazardous materials

**DOI:** 10.1016/j.ijdrr.2018.02.036

**Published:** 2018-06

**Authors:** Luca Grieco, Hazel Gleed, Stephen Groves, Simon Dyer, Martin Utley

**Affiliations:** a Clinical Operational Research Unit, University College London, 4 Taviton Street, London WC1H 0BT, United Kingdom; b National Health Service England South, York House, 18-20 Massetts Road, Horley, Surrey RH6 7DE, United Kingdom; c National Health Service England, Skipton House, 80 London Road, London SE1 6LH, United Kingdom; d UK Department of Health, Richmond House, 79 Whitehall, London SW1A 2NS, United Kingdom

**Keywords:** Allocation, Health service, Accident, Queueing, Government, Emergency preparedness

## Abstract

Accidents involving release of chemical, biological, radiological or nuclear substances may prompt the need to decontaminate exposed casualties prior to further medical treatment. Health service workers who carry out decontamination procedures wear protective suits to avoid direct contact with contaminants.

We developed an analytical framework based on queueing theory to inform UK Department of Health’s decisions on the stock of protective suits that ambulance services and hospitals with emergency departments in England should hold. Our aim was to ensure that such allocation gave an accepted degree of resilience to locally identified hazards.

Here we give an overview of our work and describe how we incorporated information in the public domain about local hazards with expert opinion about the patterns of demand for decontamination associated with different types of incident. We also give an account of how we worked with decision makers to inform national guidance on this topic.

## 1 Introduction

### 1.1 HazMat events

Incidents involving release of Chemical, Biological, Radiological, or Nuclear (CBRN) materials can have a significant social and health impact. When caused by human error, technological failure or, for example, extreme weather events, these are commonly referred to as “HazMat” events. Such accidents, as well as malicious incidents (for instance, criminal or terrorist acts), have the potential for significant human losses and environmental damage. North Atlantic Treaty Organization (NATO)’s guidelines for first response [1] give top priority to minimising the number of human deaths. In particular, healthcare workers are required to establish decontamination and triage areas and carry out decontamination procedures in order to end casualties’ exposure to the hazardous substance as soon as possible and prior to further clinical treatment. Decontamination is also fundamental to prevent the spread of toxic substances to other people/areas.

### 1.2 Our project

The work presented in this paper is focused on the problem of deciding how much personal protective equipment is needed at different points within the health system for the system to have a given degree of resilience to HazMat events. In particular, we focused on the provision of special protective suits as worn by healthcare workers during decontamination procedures. Each suit incorporates an internal respiratory system and enables full isolation of its wearer from all hazardous materials considered in this project [2].

Our objective was to determine the stock of such suits that should be held by each ambulance service and each hospital with an emergency department in England.

We were commissioned to work on this problem by the UK Department of Health (DH) through a responsive Operational Research (OR) facility that we provide to them to support health protection policy. The work was conducted in close collaboration with partners at the National Health Service (NHS) England responsible for providing national guidance to ambulance services and local hospitals. In this way NHS England, on behalf of the NHS in England, took on the role of client for the work, with DH as project sponsor.

### 1.3 Operational Research approaches in emergency preparedness

Having sufficient resources in place to cope with a range of potential events lies at the heart of emergency preparedness. NATO’s guidelines [1] emphasise the importance of such planning on the part of responding agencies, such as ambulance services, emergency departments in hospitals, police and fire brigades. However, HazMat accidents and malicious CBRN events and their consequences are intrinsically unpredictable and planning decisions need to reflect this. OR methods are particularly suitable to deal with this unpredictability, particularly in relation to informing decisions around resource allocation, taking into account risks in different areas, logistical aspects and budget limits.

Quantitative approaches to support emergency preparedness in general have been developed in the last decades. In 2006, Altay and Green [3] reviewed the literature of OR applied to disaster operations management, defining disasters as any emergency that is not an “everyday emergency”. This definition of disaster thus includes HazMat events. The main conclusion by the authors was that while several models have been conceived around disaster preparedness, there is a lack of theory development and of actual application of existing models to real-world cases. We also need a better understanding of what the inputs of such models should be, including event features. The review of Altay and Green [3] was updated in 2013 by Galindo & Batta [4], who observed a substantial progress in the development of case studies albeit with the common drawback of simplifying theoretical models by using limited and unrealistic assumptions. Works dealing with resource allocation for disaster preparedness have been published in several fields of application. Natural hazards constitute the most represented topic, including: approaches based on Stackelberg game [5] or non-linear mixed integer programming [6] to determine the optimal allocation of shelters for flood evacuation planning; two-phase approaches, i.e. preoperational (resource allocation closer to sites with higher hazards) and operational (during event), to optimise the response to wildfire (or natural hazards in general) across a region [7]; facility location models to allocate fire trucks in a geographical area in order to achieve a certain degree of zone coverage [8]. Preparedness for oil spills has also been the subject of mathematical modelling, for instance in the papers by Iakovou et al. [9] and Belardo et al. [10] dealing with optimal location/capacity of clean up equipment. Facility location models were also published for medical supplies or public services needed following (in the short or medium term) large-scale emergencies [11], [12] and to allocate ambulances in order to meet large demand volumes [8]. On the specific problem of preparedness for HazMat or CBRN events, Zaric et al. [13] studied the cost-effectiveness of different strategies for stockpiling and distributing medical supplies for response to anthrax bioterrorism, Lee et al. [14] developed systems for early detection of CBRN incidents and software tools for real-time capacity planning, Berman et al. [15] built an optimisation model to allocate limited emergency resources following identification of a bioterrorist attack on an airport. Discrete event simulation was also used to study specific scenarios of simultaneous CBRN attacks to help the Fire and Rescue Service to better allocate resources across England [16].

Lack of historical data as well as sensitivity of some information owned by decision makers around response models and procedures constitute a challenge for the development of methods to improve preparedness for HazMat events. Moreover, policy makers need to be in a position to use the mathematical models and/or interpret the results before taking decisions and spreading guidelines. Therefore, quantitative approaches towards improving preparedness to such events should be balanced between computational complexity and usability [17].

## 2 An analytical framework tailored to the national context

**Fig. 1:**
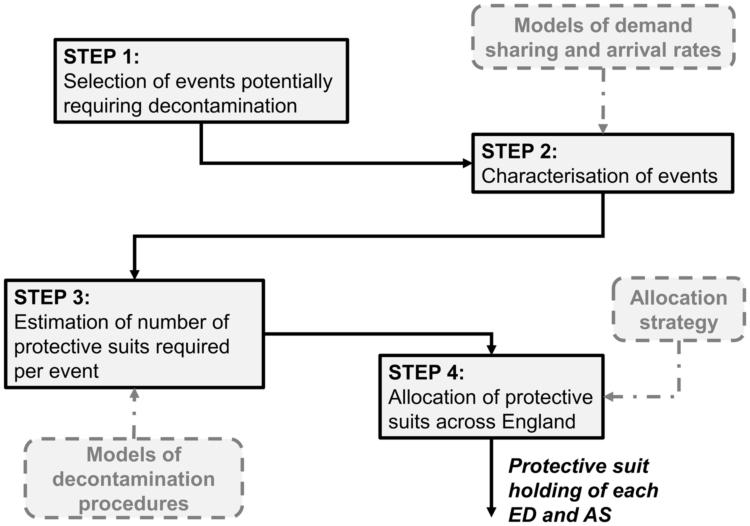
Approach followed for estimating the number of protective suits required by each ambulance service (AS) and emergency department (ED) across England.

The analytical framework we developed (see Fig. 1 for a schematic diagram) was influenced not just by the intrinsic characteristics of the problem at hand but also by the context in which we were working. Specifically, our work was influenced by client and project sponsor perspectives on the information available about potential HazMat events and by the nature of the decisions to be made. Moreover, lack of detailed information about event locations and precise estimates of event likelihood led us to follow a precautionary approach for determining the allocation of protective suits. The approach we followed consisted of four steps:

• Step 1 – We selected a list of HazMat events potentially requiring decontamination of casualties exposed to chemical, biological, radiological or nuclear materials.
• Step 2 – We used available information on the nature of these events to estimate the proportions of casualties to be accounted for by different healthcare services, along with the likely pattern of arrivals over time.
• Step 3 – We estimated the demand for protective suits for each healthcare service in response to single events occurring in a given region, based on the expected number of casualties and on the characteristics of the decontamination procedures carried out.
• Step 4 – We determined an allocation strategy allowing each healthcare service responsible for a particular (group of) region(s) to be resilient to HazMat events characterised by a minimum likelihood to happen in that(those) region(s).

In the remainder of this section we give an overview of our modelling work. Full technical details are provided as Supplementary Materials.

### 2.1 Selection of HazMat events

As a requirement of the Civil Contingencies Act introduced to UK law in 2004 [18], a Local Resilience Forum (LRF) has been formed by key emergency responders in each of the 39 police areas in England. Every LRF is required to maintain a Community Risk Register (CRR), which is a publicly available document reporting a list of accidental events potentially causing mass casualties. The description of events and their impact (health, social, economic and environmental effects) are determined centrally in the UK National Risk Register. Local Resilience Fora have the role of selecting for the CRR those events they consider relevant to them locally and attributing to them a semi-quantitative likelihood estimate by choosing one of five probability levels of each event happening in the area within the next five years. Following advice by project sponsor and client, we took the set of CRRs for England as the starting point for our analysis in order to align our work with the relevant decision processes.

From the Community Risk Registers (CRRs) we extracted a list of HazMat events (Table 1) potentially requiring decontamination. We selected those events appearing in at least one CRR across all English LRFs. Due to the absence of more detailed data about event impacts in terms of contaminated casualties, we used the “number of casualties” or “hospital admissions” reported in the event descriptions as our estimates of the number of people requiring decontamination. Local assessments of event likelihoods were also retrieved from each CRR.

**Table 1:** List of HazMat events selected from and reported in at least one Community Risk Register across English LRFs. Event type and outcome descriptions are also reported, with expected “number of casualties” or “hospital admissions” highlighted in bold.

**Type**	**Category**	**Outcome Description**
Nuclear	H10. Radioactive substance release from a nuclear reactor.	Health countermeasures during the emergency phase required up to 30 km from site with approximately 21,000 people advise to shelter and take stable iodine. No fatal deterministic health effects are anticipated, however there may be up to **2500** non-fatal effects if no countermeasures are applied.
	HL31. Limited radioactive substance release from a nuclear accident.	Up to 1 km from site causing up to 50 fatalities and **500** casualties.
Chemical	H8. Very large toxic chemical release.	Up to 10 km from site causing up to 2000 fatalities and **10000** casualties. Toxic release could be due to loss of containment of chlorine - or a number of other chemicals, e.g. anhydrous hydrofluoric acid, refrigerated ammonia, sulphur dioxide (or trioxide) gas.
	H9. Large toxic chemical release.	Up to 3 km from site causing up to 50 fatalities and up to **2000** casualties.
	HL12. Local accident involving transport of hazardous chemicals.	Up to 50 fatalities and up to **500** casualties (direct injuries from the accident would be similar to road or rail accidents; indirect casualties are possible, if substance covers wide area). The extent of the impact would depend on substance involved, quantity, nature and location of accident. The assumption is based on phosgene/chlorine.
	HL2. Localised industrial accident involving large toxic release (e.g. from a site storing large quantities of chlorine).	Up to 3 km from site causing up to 30 fatalities and up to **250** casualties.
	HL3. Localised industrial accident involving small toxic release.	Up to 1 km from site causing up to 10 fatalities and up to **100** casualties.
Biological	H12. Biological substance release from facility where pathogens are handled deliberately (e.g. pathogen release from containment laboratory).	Up to 5 fatalities and serious injuries or off-site impact requiring up to **500** hospital admissions.
	H46. Biological substance release during an unrelated work activity or industrial process (e.g. Legionella release due to improperly maintained building environmental control systems).	Up to 10 fatalities and serious injuries or off-site impact resulting in up to **1000** casualties.

Table 1 reports the HazMat events selected. Please note that only biological, chemical and nuclear events were found (no relevant radiological events were included in the CRRs of English Local Resilience Fora).

### 2.2 Characterisation of HazMat events

Depending on the nature of a HazMat event, we assumed that a proportion of contaminated casualties would remain at the site of the event to be decontaminated by the relevant ambulance service, with the remainder self-presenting at emergency departments in the LRF in the hours that follow the event (e.g. upon manifestation of symptoms). Thus, the proportions of casualties treated by the two types of service provider needed to be estimated for each event along with, for casualties attending emergency departments, a plausible pattern of arrivals over time.

#### 2.2.1 Sharing of casualties between ambulance services and emergency departments

Different factors can determine the proportion of contaminated patients that remain at the site of the event. For instance, an immediately detectable event in a place where safety procedures are well established (e.g. a chemical accident in a factory) would most likely involve only ambulance services, as all the contaminated people would be isolated and waiting for the decontamination units to be set up. On the contrary, a silent release of toxic substance in a public place would probably be detectable only after many contaminated people have already left the event scene, and consequently a proportion of casualties would need to be treated at emergency departments.

Experts from the National Ambulance Resilience Unit (NARU) advised us on the proportions of “on-site” versus “self-presenting” casualties associated with each of the HazMat events identified from analysis of CRRs.

#### 2.2.2 Sharing of casualties among healthcare services of the same type

We considered a hierarchical structure for the health system formed of two types of healthcare service providers, namely ambulance services (AS) and hospital emergency departments (ED), distributed across regions (LRFs). An ambulance service is responsible for one or more LRFs, and in every LRF there are one or more hospital emergency departments. While acknowledging that there could be cross-border sharing of resources and casualties, for the purpose of this work it was agreed with the project sponsor and client that we should assume no overlap between services (i.e. each LRF is served by a unique AS and each ED receives casualties from a unique LRF) throughout our modelling work. Therefore, in response to a HazMat event of a given type happening in a given LRF:

• On-site casualties – These would be decontaminated by the only AS responsible for that LRF.
• Self-presenting casualties – CRRs do not contain a priori information about where exactly each event would possibly take place in an LRF or about how casualties would split among EDs depending on the event, so we assumed that all EDs within the same LRF need to plan for equal proportions of casualties solely based on the density of EDs within the LRF: the higher the density, the smaller the proportion of casualties assigned to each ED. We computed such proportions using a generalised version of the logistic function and ensuring that: i) each proportion is no less than a minimum level specified by the client; ii) the sum of all proportions across hospitals in each LRF is no less than 1.

#### 2.2.3 Self-presenting arrival patterns for emergency departments

While all ambulance casualties are already on site when decontamination procedures start (therefore the number of people to treat is already known), self-presenting casualties reach hospitals during a time window specific to the event type and their arrival rate might change over time [19]. The rate at which patients arrive at a hospital following an event strongly depends on when they start to recognise the symptoms, which may vary based on the specific contaminant, or when they become aware of the incident (e.g. through mass media).

**Fig. 2:**
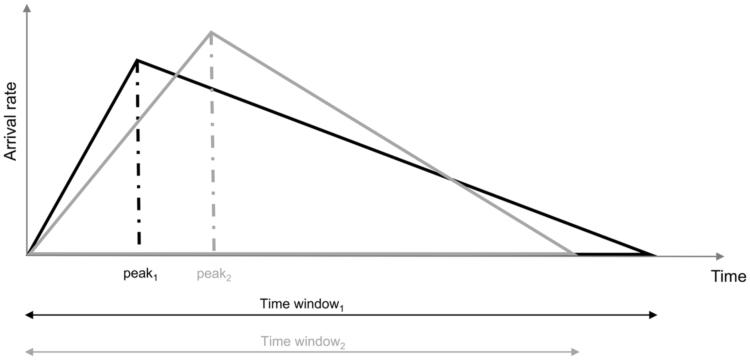
Illustrative examples of triangle-shaped functions used to model time-varying arrival rates of casualties at EDs. *Note:* the actual parameters used in the analysis constitute sensitive information and cannot be disclosed in this paper.

Kilic et al. [19] used a modified gamma probability density function to model time-dependent arrival rates. However, due to the difficulties in parameterising this function in the absence of historical data, we decided to use a triangle-shaped function (Fig. 2), fully defined by two parameters:

• time window, over which patients arrive at the ED for decontamination;
• peak time, at which the arrival rate reaches its maximum.

Values for these two parameters were agreed for each event type (chemical, biological, nuclear) with our collaborators from the UK Department of Health and NHS England who have extensive experience of planning related to HazMat and CBRN incidents.

### 2.3 Single-event demand estimation

Decontamination of casualties exposed to CBRN materials is carried out in decontamination units (usually tents) that are assembled as required. We relied upon the following assumptions based on existing, official guidelines regarding the deployment of decontamination units in the UK:

• each healthcare service owns a (potentially different) number of decontamination units, that can be deployed in parallel if necessary;
• each decontamination unit is operated by a team formed of trained healthcare workers wearing protective suits – team size is fixed across healthcare services of the same type;
• each decontamination unit can support a number of patient lanes sharing the same team of healthcare workers – a lane can be “activated” or “deactivated” depending on the number of casualties waiting to be decontaminated at a given time;
• each team operates for an amount of time (fixed across healthcare services of the same type) corresponding to a “decontamination session”, after which they need to be replaced by another team – all members of the same team are assumed to start and end their session at the same time;
• decontamination sessions cannot be interrupted once they start;
• at the end of a decontamination session, the decontamination unit is used for team “self-decontamination” (needed before taking the protective suit off), during which the team split into sub-groups each occupying a lane for a given amount of time – as one or more lanes become available again they can be used by the following team for the next decontamination session;
• decontamination of a patient is a standard procedure consisting of removing contaminants from their body with the help of water and soap – time needed to decontaminate a patient is influenced by the number of active lanes in the decontamination unit (as all lanes share the same team).

In order to determine single-event demand estimates, we modelled the decontamination process as a queueing system with a single queue and as many servers as the total number of lanes used. We used different models for ambulance services and for emergency departments, given the different ways patients present to them for decontamination.

#### 2.3.1 Single-event demand estimation for ambulance services

Decontamination by ambulance services is characterised by the fact that all contaminated people are at the site of the HazMat event. We assumed that enough staff members are available to run decontamination sessions until all patients are cleared.

**Fig. 3:**
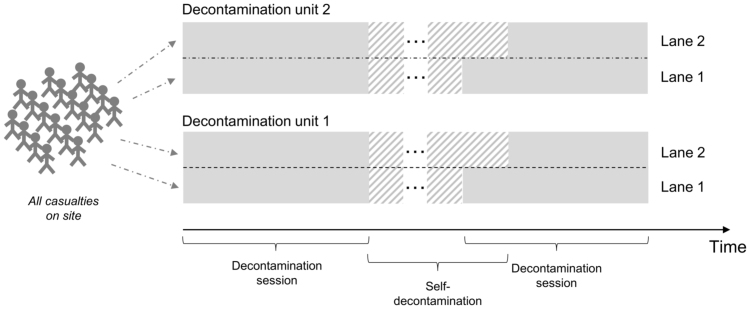
An illustration of our algorithm for ambulance services. *Note:* while the last group of team members is undergoing self-decontamination, the following teams can use only one lane of each decontamination unit for decontaminating patients, with the total service rate of the unit being different from when both lanes are used.

We modelled this process as a queueing system with the initial queue sized as the number of on-site casualties associated with the given HazMat event. We developed a simple algorithm (whose functioning is summarised in Fig. 3) to estimate the number of decontamination sessions needed, and thus of protective suits. In principle, at the beginning of the decontamination procedures, decontamination units are activated in parallel in order to cover as many patients as possible during the first set of sessions (we suppose here that all the parallel sessions start at the same time). After the first set of sessions ends, team self-decontamination takes place and new sessions will start, if needed, as soon as lanes become available again.

#### 2.3.2 Single-event demand estimation for emergency departments

Staff members in an emergency department may not be aware of the type of HazMat event when contaminated people start self-presenting. Moreover, as described previously, the arrival of contaminated patients at an emergency department following a HazMat event may be delayed in time, with a non-constant arrival rate. It is then nearly impossible for healthcare workers to have an estimate of the total number of people that will need to be treated, and when. For such scenarios it was considered reasonable to assume that emergency departments will adapt their decontamination capacity (i.e. number of units/lanes to run in parallel), depending on the current number of people waiting for treatment.

We modelled the decontamination process at emergency departments as a queueing system and adapted methods from queueing theory literature to estimate the number of decontamination sessions that need to be carried out, and thus of protective suits needed, following a HazMat event of a given type.

The process can be modelled as a M(t)/M(t)/c(t) queueing system [20]:

• we assume time-dependent Poisson arrivals for contaminated patients;
• service time (i.e. time to decontaminate each patient) is assumed to be exponentially distributed, with the mean service time dependent on the number of lanes in use;
• the current capacity of the system corresponds to the total number of active lanes and is state-dependent, with scope for activating or deactivating lanes in response to queue size.

Solution of the corresponding ordinary differential equations would be very challenging given the complexity and dynamic nature of this system. We thus developed an algorithm providing an approximation of its behaviour in time at very little computational cost. This choice was also motivated by us needing to be able to re-run the entire framework described in this paper with very short notice, following possible requirements from our client about parameter changes.

Let us define time intervals $(t_{k-1},t_{k}],k=1,\ldots ,T$, with $t_{T}=$ end of decontamination procedures. Please note that there might be cases where decontamination is not required after a certain amount of time has elapsed since a HazMat event, corresponding, say, to people having changed clothes or showered prior to experiencing symptoms. Therefore, for a given event involving self-presenters at emergency departments, decontamination procedures are assumed to cease at a given time instant $t_{T}$.

**Fig. 4:**
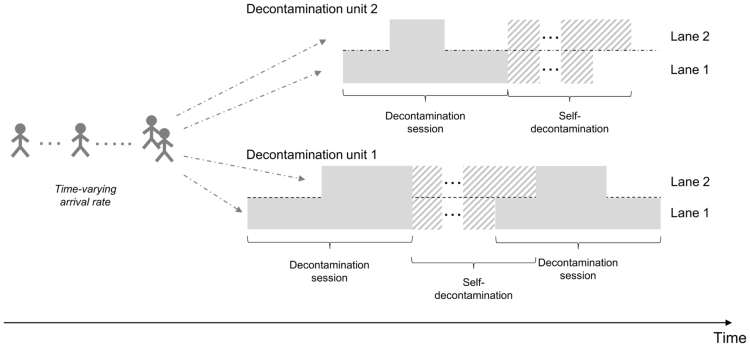
An illustration of the algorithm for emergency departments. *Note:* lanes (and decontamination units) can be activated or deactivated depending on the current number of casualties queueing, with service rates depending on the number of active lanes in each decontamination unit.

In our algorithm, decisions about system capacity are assumed to be taken at the end of each time interval. We rely on the realistic assumption that the capacity (and consequently the service time) of the system can be modified during the incident depending on the current number of patients in the queue (see Fig. 4 for an illustrative example). In particular, an additional lane is activated when the current number of active lanes is not sufficient to treat the current number of patients within ongoing sessions, whereas it is deactivated at the end of a session if a smaller capacity would be sufficient to clear all patients currently queueing.

The estimated queue length $q_{k}$ (at the end of time interval k) is computed using a method originally proposed by Raik Stolletz [20]: the M(t)/M(t)/c(t) system is approximated with a particular (stationary) $M_{k}/M_{k}/c_{k}/c_{k}$ system during time interval $\left(t_{k-1},t_{k}\right)$ and the number of “blocked” patients is carried over into time interval $\left(t_{k},t_{k+1}\right)$. We adapted this method to the case of heterogeneous servers by using the generalisation of the Erlang-loss formula proposed by Saglam and Shahbazov [21].

Our algorithm also computes $r_{k}$, defined as the number of patients that could be potentially decontaminated if system capacity at the end of time interval $\left(t_{k-1},t_{k}\right)$ is kept until the end of all ongoing sessions.

Decisions are taken based on the following rules:

• If $q_{k}=r_{k}$, then no action is taken (the current capacity is kept for the next time interval).
• If $q_{k}>r_{k}$ and at least one lane is inactive in the system, then a lane is chosen to be activated. Lanes are iteratively activated until $q_{k}\leq r_{k}$.
• If $q_{k}<r_{k}$ and at least one lane is active in the system, then a lane is chosen to be deactivated. But if that lane is the only active one in its decontamination unit, then it is deactivated only if the corresponding decontamination session ends exactly at the current time point (decontamination sessions cannot be interrupted once they start). Lanes are iteratively deactivated ensuring that $q_{k}$ never exceeds $r_{k}$.

### 2.4 Allocation strategy

The last step of our work consisted in determining protective suit demand estimates across all events, for each healthcare service provider. We used a “plausible worst-case scenario” approach: the required stock of protective suits to be held by a healthcare service responsible in a given region corresponding to the maximum single-event demand estimates for that healthcare service across all events with likelihood level equal to or above a given threshold in that region.

## 3 Example of application of the framework

**Fig. 5:**
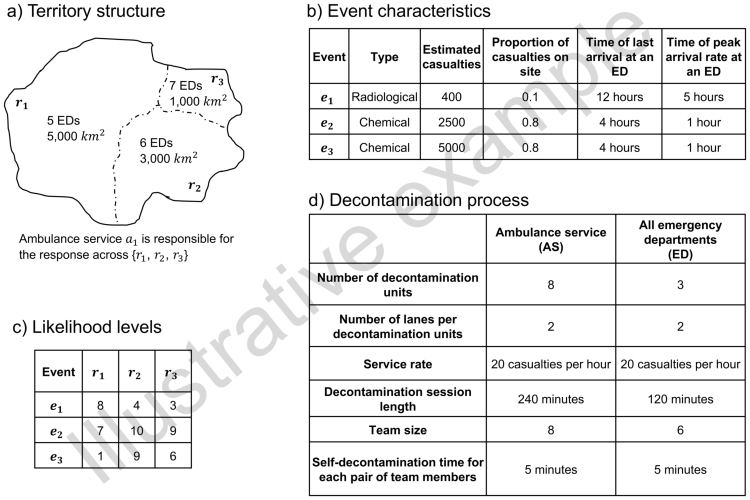
Example of model input. a) We consider a hypothetical region formed of three regions {$r_{1},r_{2},r_{3}$} of different sizes and with different numbers of emergency departments (ED). A single ambulance service $a_{1}$ is assumed to be responsible for the three regions. b) We also consider three events {$e_{1},e_{2},e_{3}$} with different features. c) We assume that a discretised likelihood level, ranging from 1 to 10 (1 = ”very unlikely” and 10 = “very likely”), has been determined for each event to happen in each region. d) Decontamination procedures by ambulance services and emergency departments have different characteristics. We further assume that each team member requires 1 suit per decontamination session.

In this section, we illustrate an example of application of our framework to a hypothetical region. Fig. 5 summarises all assumptions made in this example.

Suppose we want to determine the number of protective suits for each emergency department and for the ambulance service to ensure their resilience to any event of likelihood level 7 or above (i.e. L=7). Table 2 reports the number of casualties associated with each service, computed using our framework. These are obtained starting from the event-specific estimated casualties and proportion of casualties on site reported in Fig. 5b, as well as (for emergency departments) from the region characteristics shown in Fig. 5a. For instance, the number of casualties for the ambulance service following event $e_{1}$ (i.e. 40) is obtained by multiplying the event-specific estimated casualties (i.e. 400) by the event-specific proportion of casualties on site (i.e. 0.1). Then, the corresponding global number of casualties for emergency departments (in any region) is 360. By multiplying this number by the region-specific coefficient (based on the regional density of emergency departments), we obtain the number of casualties for each emergency department following event $e_{1}$.

**Table 2:** Number of casualties per service, per event. The second column refers to the number of casualties the ambulance service would need to plan for should the corresponding event happen. The third column refers to the number of casualties associated with each emergency department of region $r_{i}$. For instance, each of the 6 emergency departments in region $r_{2}$ would need to plan for 322 casualties should event $e_{1}$ happen.

Events	Casualties associated with ambulance service $a_{1}$	Casualties associated with each ED of region $r_{i}$		
		$r_{1}$	$r_{2}$	$r_{3}$
$e_{1}$	40	343	322	219
$e_{2}$	2000	477	447	304
$e_{3}$	4000	953	893	608

Step 3 of our framework enables computation of the number of decontamination sessions required per event, respectively for every emergency department in each region and for the ambulance service, starting from the number of casualties per service given in Table 2. For each event, the time-varying arrival rate function is parameterised using data from the last two columns in Fig. 5b. Decontamination process features are taken from Fig. 5d. Results obtained for our example are summarised in Table 3.

**Table 3:** Required decontamination sessions per service, per event, obtained with our framework for emergency departments and ambulance services, respectively. The number of protective suits required is easily determined by multiplying the number of sessions by the corresponding team sizes (8 for the ambulance service and 6 for the emergency departments). Highlighted in bold are results corresponding to likelihood levels of 7 and above (cf. Fig. 5c).

Events	Decontamination sessions for ambulance service $a_{1}$	Decontamination sessions for each ED of region $r_{i}$		
		$r_{1}$	$r_{2}$	$r_{3}$
$e_{1}$	**1**	**9**	8	7
$e_{2}$	**16**	**8**	**8**	**6**
$e_{3}$	**32**	16	**15**	10

According to our allocation strategy, each emergency department would need to account for the plausible worst-case scenario in terms of number of sessions/protective suits required among the events of likelihood level 7 or above. Therefore:

• Each of the 5 emergency departments in region $r_{1}$ would need to plan for max{9,8}=9 decontamination sessions, giving a total of 9×6 = 54 suits (as 6 suits are used per session). In this case, the total number of suits for the region is 54×5 = 270 (as there are 5 emergency departments in the region).
• Each of the 6 emergency departments in region $r_{2}$ would need to plan for max{8,15}=15 decontamination sessions (i.e. 15×6 = 90 suits). Total number of suits for the region: 90×6 = 540.
• Each of the 7 emergency departments in region $r_{3}$ would need to plan for max{6}=6 decontamination sessions (i.e. 6×6 = 36 suits). Total number of suits for the region: 36×7 = 252.

Ambulance service $a_{1}$ would need to plan for the plausible worst-case scenario in terms of number of sessions/protective suits required among the events of likelihood level 7 or above in at least one of the three regions. Since all events satisfy this condition, $a_{1}$ would need to plan for max{1,16,32}=32 decontamination sessions, giving a total of 32×8 = 256 suits (as 8 suits are used per session).

Please note that how our notion of “plausible worst-case scenario” is in terms of the number of protective suits required in response to an event, and not in terms of number of casualties triggered by that event. For instance, in region $r_{1}$, event $e_{1}$ is associated with 343 casualties and requires 9 decontamination sessions for emergency departments, whereas event $e_{2}$ is associated with a much higher number of casualties (i.e. 477) but a lower number of sessions required (i.e. 8). This is due to the different pattern of arrivals in the two cases ($e_{1}$ is characterised by a much longer time window than $e_{2}$ during which patients arrive at hospitals) and the fact that decontamination sessions cannot be interrupted once they start.

**Fig. 6:**
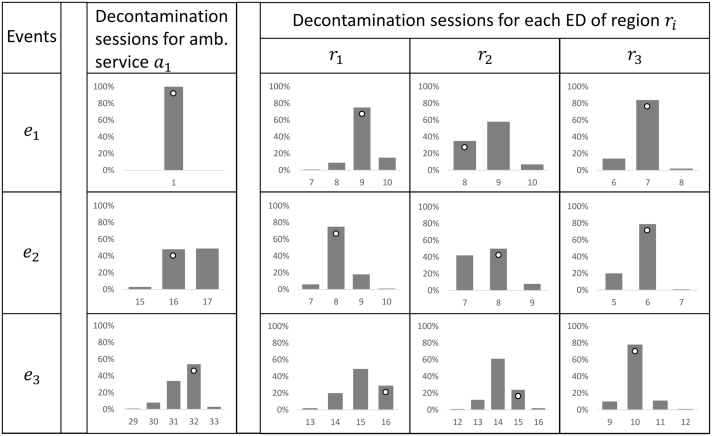
Simulation results. Each histogram shows the distribution of number of sessions (horizontal axis) obtained from our simulator, across 100 runs. The results obtained from our analytical framework (cf. Table 3) are denoted by white dots.

For an internal validation of our approach, we compared the results obtained for this illustrative example (using our modified version of Stolletz’s method) with those obtained from a simulator that we developed and implemented in R programming language (code available upon request). The simulator accounts for variability in service time and time for self-decontamination, both modelled as exponentially distributed stochastic variables. For emergency departments, arrivals are obtained according to a Poisson process with time-varying arrival rate. The results (number of decontamination sessions needed) obtained from the analytical model are very close to the most frequent results obtained from the simulator (Fig. 6).

## 4 Informing national guidelines

With our approach, the required stock level of protective suits (for a hospital in an LRF or for an ambulance service responsible for a set of LRFs) is estimated by computing the biggest number of suits needed across all events with likelihood level above or equal to a given threshold in the corresponding region. Using this as the actual numbers of protective suits to be held by healthcare services may be not reasonable if considering the fact that a second event might happen in the same place before the appropriate stock of protective suits is replenished: healthcare services might not be able to respond to that second event.

Therefore, we provided our client with results for different likelihood threshold levels that they used as “building blocks”, that is, with allocation of protective suits determined by summing up demand estimates obtained for different scenarios of interest (e.g. planning for the plausible worst-case scenario with likelihood level above or equal to L plus another plausible worst-case scenario with likelihood level above or equal to L+1).

Our analysis fed into guidance issued by NHS England to ambulance services and hospitals with emergency departments, directly informing decisions valued at millions of GBP.

## 5 Discussion

We developed an analytical framework allowing estimation of the number of protective suits needed to be held by each ambulance service and emergency department within a given region in order to be able to carry out decontamination procedures in response to HazMat events. Our approach consisted first of gathering publicly available information about local risks and event features, and formally modelling the relevant processes in collaboration with specialists from NHS England, the UK Department of Health and the National Ambulance Resilience Unit (NARU). Algorithms based on queueing theory were then developed to obtain expected protective suit demands for each event considered. Finally, an allocation strategy based on “plausible worst-case scenario” selection was adopted to suggest protective suit holdings for each healthcare service considered. Our results fed into official guidance issued by NHS England to ambulance services and hospitals with emergency departments in England.

Several simplifying assumptions were made throughout this work. Most of them were directly agreed with our client. These were all assumptions around the decontamination process and event features (e.g. average service time, session lengths, delays for self-decontamination, event time windows, peaks of arrivals, etc.), and were mainly derived from official guidelines, augmented by expertise of our collaborators. We further made some technical assumptions, including the generalised logistic function to determine sharing of casualties among emergency departments, the shape of the arrival rate function, the probability distributions of arrivals and of service times. These choices were reasonably, though arbitrarily, derived from discussions with experts and literature searching. Our framework could easily be used with different parametrisations should empirical data become available or if decision makers wanted to alter the planning assumptions in the future. For instance, planning for scenarios characterised by the presence of particular contaminants or by extreme weather conditions might require changes to assumptions about service times (due to healthcare workers’ performance) and/or the modes of operation of the protective suits. (e.g. due to very low/high temperatures within the suit, a need for replacing suit’s air filter canisters during a decontamination session, etc.).

We followed a modular approach composed of independent, sequential steps. Each step (depicted in Fig. 1) can be refined independently of the others and adapted to other contexts. In this respect, it is important to note that some of our modelling choices were attuned to the specific decision being informed. This work was restricted to the case of protective suits for use in decontaminating exposed casualties prior to any form of medical treatment. In other circumstances, more complex medical procedures involving drug administration [22] and/or advanced life support interventions [2], [23] might be considered for incorporation into the model of single-event demand estimation. The framework could also be easily extended to inform decisions involving provision of different types of protective equipment.

At the request of our clients, we did not incorporate in our estimates the potential for sharing of resources across regional boundaries or between organisations within a region. While there would be some scope for sharing of resources in some circumstances, the view taken was that the resilience of organisations and regions should not be dependent on informal arrangements of this nature. The potential for formal pooling of resources across regions through the use of strategic storage facilities may form the basis of future work.

Another limitation of our work was that we did not estimate any additional number of suits required to ensure that the size distribution of suits purchased adequately matches the staff the organisation would want to deploy in a given event. We discussed this issue with the client early in the project and it was decided that any adjustment to the number of suits purchased should be made locally using knowledge of the local staff trained in using the suits and training policies.

We are aware that considering detailed geographical information about locations of healthcare services and potential locations of HazMat events would certainly lead to different and more accurate demand estimates. Furthermore, as we mentioned above, we have only considered accidental events so far. Malicious events were excluded from our work due to high sensitivity of information about the likelihood, potential targets and potential impacts of such events. Ideally they should be included in the analysis for a more reliable estimation of the need for protective suits. This problem could be tackled by developing software to be used independently by decision makers, in order to allow safe inclusion of sensitive input data. Also, assumptions around staff performance may need to differ for malicious event. While we note that healthcare workers providing support to casualties from accidental HazMat accidents are subject to high levels of stress, being at risk of contamination themselves and being exposed to a number of limiting environmental factors (e.g. heat, noise, multiple casualties) [2], response to malicious events might be characterised by increased stress levels and the perceived threat experienced by healthcare workers and casualties. This constitutes an additional challenge to modelling of workflows as actual operations might deviate substantially from planned procedures.

Finally, our work has the potential to be extended to other agencies, namely police and fire & rescue who have a role in decontamination, as well as to the other UK Countries, who have already shown their interest in the approach followed.
