# Supplementary material for: Informing decisions on the purchase of equipment used by health services in response to incidents involving hazardous materials
Source: Int J Disaster Risk Reduct. 2018 Jun;28:113–21. doi: 10.1016/j.ijdrr.2018.02.036 (PMC7707622; doi:10.1016/j.ijdrr.2018.02.036)
Supplement: Supplementary file 1 — Supplementary material [file mmc1.docx]

**SUPPLEMENTARY MATERIAL (all following sections – to be considered as a single, pfd document)**

**Technical documentation**

In this document, we report formal assumptions and technical details regarding our framework for estimating the number of protective suits required by each ambulance service and emergency department across England.

**1. Notation**

Health system

We consider a hierarchical structure for the health system formed of two types of healthcare service providers, namely ambulance services (AS) and hospital emergency departments (ED), distributed across regions (LRFs). An ambulance service is responsible for one or more LRFs, and in every LRF there are one or more hospital emergency departments. We assume that there is no overlap between services (i.e. each LRF is served by a unique AS and each ED receives casualties from a unique LRF). Let us define the following:

- $A$ is the set of ambulance services;
- $R_{a}$ is the set of all LRFs that ambulance service $a$ is responsible for, with $a\in A$;
- $H_{r}$ is the set of emergency departments within LRF $r$ (for any $r\in R_{a}$ and $a\in A$).

Events

We denote with $E$ the set of HazMat events potentially requiring decontamination and defined nationally. Let $z^{e}$ be the estimated number of contaminated casualties associated with event $e\in E$. Each event belongs to one of the following types: $\Omega=$ {chemical, biological, radiological, nuclear}. Events are also characterised by a likelihood level. In the following, we will denote with $p_{r}^{e}$ the level of likelihood of event $e$ happening in LRF $r$.

We also consider the following time points relevant to each event type $\omega\in\Omega$:

- time $t_{0}^{\omega}$ at which any event of type $\omega$ happens;
- time $t_{N}^{\omega}$ at which the last contaminated casualty arrives at an ED, following any event of type $\omega$;
- time $t_{M}^{\omega}$, characterised by the highest arrival rate (peak) at an ED within the period $\left[ t_{0}^{\omega},t_{N}^{\omega} \right]$, following any event of type $\omega$.

Demand for decontamination

- Let $z_{A}^{e}$ be the number of casualties decontaminated on scene by an ambulance service and $z_{H}^{e}$ be the number of self-presenting contaminated casualties to be treated by emergency departments, following event $e$.
- $n_{a}^{e}$ denotes the number of casualties that ambulance service $a$ should plan for and $n_{h}^{e}$ is the number of casualties that emergency department $h$ should plan for, to be resilient to event $e$.

Decontamination process

We assume that decontamination is carried out in decontamination units (usually tents) that are assembled as required. We model the decontamination process as follows.

- Let $L_{a}$ be the set of decontamination units owned by ambulance service $a\in A$. Let $L_{h}$ be the set of decontamination units owned by emergency department $h\in H_{r}$ (for any $r\in R_{a}$ and $a\in A$).
- More than one decontamination unit can be deployed at a time, up to $\left| L_{a} \right|$ or $\left| L_{h} \right|$, respectively.
- A team formed of a number $m_{A}$ or $m_{H}$ (for any AS or ED, respectively) of staff members wearing protective suits is assumed to carry out decontamination using a decontamination unit.
- Any AS or ED team can operate for a maximum amount of time ${\Delta t}_{A}$ or ${\Delta t}_{H}$, respectively, corresponding to a decontamination session. All members of a team are assumed to start and end their session at the same time.
- Each decontamination unit $l$ (with $l\in L_{a}$ or $l\in L_{h}$) can support $w_{l}$ lanes. Each lane has an independent flow of patients, but each operating team is shared between all the lanes of a unit active at a given time.
- A lane can be activated or deactivated, depending on the number of casualties waiting to be decontaminated at a given time. We denote with $b_{l}\left( t \right)$ the number of active lanes in decontamination unit $l$ at a given time.
- At the end of a session, the decontamination unit is used for team self-decontamination. The team is assumed to split in sub-groups of $\eta$ members, each occupying a lane for a given amount of time $\delta t$. Let ${\Delta t}_{off}^{l}$ be the amount of time a decontamination unit $l$ is completely busy for self-decontamination. As one or more lanes become available again they can be used by the following team for the next patient decontamination session.
- $\mu$ denotes the service rate (patients/hour) of a lane in a decontamination unit when no other lanes are active in that unit. The total service rate of a decontamination unit $l$ is dependent on this rate and on the number of active lanes, according to the function $\Pi\left( \mu,b_{l} \right)$ defined in Appendix A.

Finally, let $s_{a}^{e}$ and $s_{h}^{e}$ be the number of decontamination sessions required, respectively, by ambulance service $a$ and emergency department $h$ following event $e$. Correspondingly, $d_{a}^{e}$ and $d_{h}^{e}$ represent the numbers of protective suits required by each healthcare service provider in response to each event.

**2. Characterisation of HazMat events**

***2.1. Sharing of casualties between ambulance services and emergency departments***

The estimated numbers of casualties that remain on site and that present at emergency departments following event $e$ are given, respectively, by $z_{A}^{e}=\left\lceil\gamma_{e}\cdot z^{e} \right\rceil$ and $z_{H}^{e}=\left\lceil(1-\gamma_{e})\cdot z^{e} \right\rceil$, where $\gamma_{e}\in\left[ 0,1 \right]$ is the proportion of patients remaining on scene following event $e$.

***2.2. Sharing of casualties among healthcare services of the same type***

Ambulance services

We assume that:

- each AS is responsible for the response across a set of LRFs;
- each AS only needs to be resilient to events happening within its corresponding LRFs (i.e. there is no overlap between ambulance service areas);
- each LRF is served by a unique AS.

Consequently, the number of casualties that ambulance service $a$ should plan for if event $e$ happens in its area, is $n_{a}^{e}\equiv z_{A}^{e}$.

Emergency departments

In general, a single LRF has more than one emergency department on its territory. Starting from $z_{H}^{e}$ we need to establish the number of patients that each ED in an LRF should plan for, which is in general dependent on geographical and demographic features.

We define the number of contaminated casualties that emergency department $h$ should plan for if event $e$ happens as $n_{h}^{e}=\left\lceil\alpha_{h}^{e}\cdot z_{H}^{e} \right\rceil$, where $\alpha_{h}^{e}\in(0,1]$ is chosen in order to ensure that at least 100% of the regional ED contaminated casualties are covered for any event (i.e. $\sum_{h\in H_{r}} n_{h}^{e}\geq z_{H}^{e}$). Further details on how $\alpha_{h}^{e}$ is computed specifically for our case study can be found in Appendix B.

***2.3. Self-presenting arrival rates for emergency departments***

It is reasonable to assume that the rate of patient arrivals at medical facilities is not constant after a HazMat event (Kilic et al., 2014). The rate at which patients arrive at a hospital following an event strongly depends on when they start to recognise the symptoms, which may vary based on the specific contaminant or when they become aware of the incident (e.g. through mass media). Modelling this aspect is important because a key assumption in our work is that decontamination sessions are of fixed length and therefore, while estimating the number of protective suits required, we need to account for the possibility of idle time during a decontamination session.

Kilic et al. (2014) used a modified gamma probability density function to model time-dependent arrival rates. However, due to the difficulties in parameterising (shape and scale factors) this function, we decided to use the triangular distribution function, defined as follows (cf. Figure 2 in main text):

|  | $\varphi_{\omega}\left( t \right)=\left\{ \begin{aligned} \frac{2 (t-t_{0}^{\omega})}{(t_{N}^{\omega}-t_{0}^{\omega})(t_{M}^{\omega}-t_{0}^{\omega})}, if t_{0}^{\omega}\leq t\leq t_{M}^{\omega} \\ \frac{2 (t_{N}^{\omega}-t)}{(t_{N}^{\omega}-t_{0}^{\omega})(t_{N}^{\omega}-t_{M}^{\omega})}, if t_{M}^{\omega}\leq t\leq t_{N}^{\omega} \\ 0, otherwise \end{aligned} \right.$ | (1) |
| --- | --- | --- |

We can then define the arrival rate function, representing the instantaneous arrival rate for an emergency department $h$ following event $e$ of type $\omega$:

|  | $\lambda_{h}^{e,\omega}\left( t \right)=n_{h}^{e}\cdot\varphi_{\omega}\left( t \right)$. | (2) |
| --- | --- | --- |

So, the expected number of self-presenters arrived within a period $\left[ t_{1},t_{2} \right]$ for emergency department $h$ following event $e$ of class $\omega$ is given by:

|  | $\Lambda_{h}^{e,\omega}\left( t_{1},t_{2} \right)=n_{h}^{e}\cdot\Phi_{\omega}\left( t_{1},t_{2} \right)$ | (3) |
| --- | --- | --- |

where

|  | $\Phi_{\omega}(t_{1},t_{2})=\int_{t_{1}}^{t_{2}} \varphi_{\omega}\left( t \right)dt$. | (4) |
| --- | --- | --- |

As $\varphi_{\omega}\left( t \right)$ is a probability distribution function, we also have $\Phi_{\omega}(t_{0}^{\omega},t_{N}^{\omega})=1$. Therefore, the integral of $\lambda_{h}^{e,\omega}\left( t \right)$ between $t_{0}^{\omega}$ and $t_{N}^{\omega}$ corresponds to the expected number of self-presenting people at emergency department $h$ following event $e$ of type $\omega$:

|  | $\Lambda_{h}^{e,\omega}\left( t_{0}^{\omega},t_{N}^{\omega} \right)=n_{h}^{e}$. | (5) |
| --- | --- | --- |

Different types of HazMat events can lead to different arrival patterns at emergency departments. The triangular distribution-based arrival rate function $\lambda_{h}^{e,\omega}\left( t \right)$ allows us to classify such events based on three parameters only: $n_{h}^{e}$, $t_{M}^{\omega}$ and $t_{N}^{\omega}$ (with event $e$ being of type $\omega$). By convention, we assume $t_{0}^{\omega}=t_{0}=0$ always. Please note that $n_{h}^{e}$ depends on the specific event and on the specific emergency department considered, whereas $t_{M}^{\omega}$ and $t_{N}^{\omega}$ depend solely on the type of event.

**3. Estimation of single-event demand for protective suits**

We modelled the decontamination process as a queueing system with a single queue and as many servers as the total number of lanes used. The algorithm described in Section 3.1 computes the number of decontamination sessions $s_{a}^{e}$ that would be required by ambulance service $a\in A$ following event $e$. This quantity is dependent on the configuration of decontamination units owned by the AS and on $n_{a}^{e}$. The algorithm described in Section 3.2 computes the number of decontamination sessions $s_{h}^{e}$ that would be required by emergency department $h$ following event $e$ of type $\omega$. This number is dependent on the configuration of decontamination units owned by the ED and on the arrival rate function $\lambda_{h}^{e,\omega}\left( t \right)$. The numbers of protective suits needed ($d_{a}^{e}$ and $d_{h}^{e}$) are computed by multiplying the numbers of decontamination sessions ($s_{a}^{e}$ and $s_{h}^{e}$, respectively) by the corresponding team size.

***3.1. Model of decontamination process for ambulance services***

Decontamination by ambulance services is characterised by the fact that all contaminated people are at the site of event $e$. We assume that enough staff members are available to run decontamination sessions until all patients are cleared.

We further rely on the following assumptions:

- an ambulance service might own decontamination units with different numbers of lanes;
- on need, decontamination units can be deployed in parallel.

Algorithm S1 enables estimation of the number of protective suits needed by a generic ambulance service $a$ (owning $\left| L_{a} \right|$ decontamination units) to clear $n_{a}^{e}$ casualties. In principle (Figure S1), at the beginning of the decontamination procedures, decontamination units are activated in parallel in order to cover as many patients as possible during the first set of sessions (we suppose here that all the parallel sessions start at the same time). After the first set of sessions ends, team self-decontamination takes place and new sessions will start, if needed, as soon as lanes become available again.


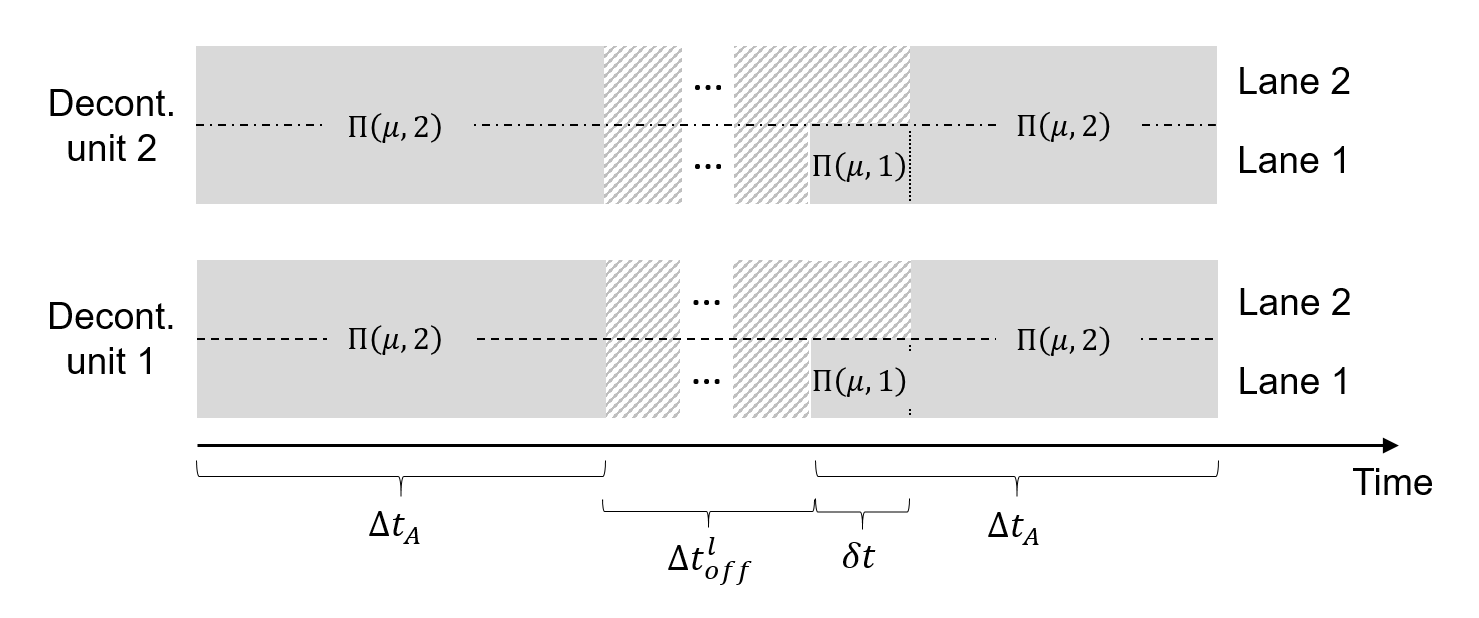


**Figure S1.** An illustration of decontamination process for ambulance services. We show here the deployment of two identical decontamination units for two sessions of time length ${\Delta t}_{A}$, assuming that self-decontamination is carried out at the end of each session with sub-groups of the team occupying a whole unit for an amount of time ${\Delta t}_{off}^{l}$. In this example, while the last group of team members is undergoing self-decontamination, only one lane of a decontamination unit can be used by the following team for decontaminating patients, with the total service rate of the unit being different from when both lanes are used (cf. Appendix A).

Let us first introduce the following quantities:

- the number of patients that can be decontaminated per session in unit $l$ when all lanes are always available is given by:

|  | $r_{full}^{l}=\Pi\left( \mu,w_{l} \right)\cdot{\Delta t}_{A}$ | (6) |
| --- | --- | --- |

- the following expression gives the number of patients decontaminated per session in unit $l$ if $o_{l}$ lanes are busy during time period $\delta t$ due to team self-decontamination (cf. Appendix C), and then all lanes are available for the remaining time (cf. Figure S1):

|  | $r_{self}^{l}=\Pi\left( \mu,w_{l}-o_{l} \right)\cdot\delta t+\Pi\left( \mu,w_{l} \right)\cdot({\Delta t}_{A}-\delta t)$ | (7) |
| --- | --- | --- |

Please note that, given the nature of the decontamination process for ambulance services, we do not explicitly compute the amount of time (${\Delta t}_{off}^{l}$) during which all lanes of a decontamination unit are used for self-decontamination, as this will not influence the number of sessions needed to clear the $n_{a}^{e}$ patients (i.e. the number of patients treated during that period is 0).

| **INITIALISATION** |
| --- |
| - $r:=n_{a}^{e}$ - $s_{a,1}^{e}=s_{a,2}^{e}=s_{a,3}^{e}:=0$ (numbers of decontamination sessions required in each of the following phases) - $l\in L_{a}$ ordered (arbitrarily) by decreasing number of units ($w_{l}$) - Compute $r_{full}^{l}$ and $r_{self}^{l}$ for each $l\in L_{a}$ |
| **PHASE 1** |
| $For l=1 to \left\vert L_{a} \right\vert$  $If (r\leq0) then GO TO$ **ENDING**  $Else:$  $s_{a,1}^{e}:=s_{a,1}^{e}+1$  $r:=r-r_{full}^{l}$ |
| **PHASE 2** |
| $If (r\leq0) then GO TO$ **ENDING**  $Else:$  $s_{a,2}^{e}:=\left\lfloor\frac{r}{\sum_{l\in L_{a}} r_{self}^{l}} \right\rfloor\cdot\left\vert L_{a} \right\vert$  $r:=r-\frac{s_{a,2}^{e}}{\left\vert L_{a} \right\vert}\cdot\sum_{l\in L_{a}} r_{self}^{l}$ |
| **PHASE 3** |
| $For l=1 to \left\vert L_{a} \right\vert$  $If (r\leq0) then GO TO$ **ENDING**  $Else:$  $s_{a,3}^{e}:=s_{a,3}^{e}+1$  $r:=r-r_{self}^{l}$ |
| **ENDING** |
| $s_{a}^{e}:=s_{a,1}^{e}+s_{a,2}^{e}+s_{a,3}^{e}$ |

**Algorithm S1.** Algorithm to compute the number of decontamination sessions required by ambulance service $a$ in response to event $e$.

The first phase corresponds to what happens at the beginning of the decontamination procedures. Decontamination units are activated in parallel in order to cover as many patients as possible during the first set of sessions (we suppose here that all the parallel units start at the same time). Note that if $s_{a,1}^{e}<\left| L_{a} \right|$ then there is no need to go on with the next phases as all patients would be decontaminated during this phase.

The second phase starts when the first set of parallel sessions ends. Now, the service rate of each unit needs to account for team self-decontamination. The quantity $\left\lfloor\frac{r}{\sum_{l\in L_{a}} r_{self}^{l}} \right\rfloor$ represents the number of sets of parallel sessions to be carried out deploying all the available decontamination units. The total number of sessions in this phase is given by multiplying this quantity by the number of decontamination units. Note that $s_{a,2}^{e}>0$ if and only if all the available decontamination units are deployed in parallel in both phases 1 and 2.

The third phase accounts for the final part of the decontamination procedure. $s_{a,3}^{e}>0$ if and only if the number of patients remaining from the second phase does not imply use of all available decontamination units in parallel. $s_{a,3}^{e}$ thus corresponds to the smallest number of units that can be run in parallel for one session in order to clear these remaining patients.

***3.2. Model of decontamination process for emergency departments***

The key idea of our algorithm is based on the realistic assumption that the capacity (and consequently the service time) of the system can be modified during the decontamination procedure depending on the current number of patients in queue (Figure S2). In particular, an additional lane is “activated” when the current number of active lanes is not sufficient to treat the current number of patients within ongoing sessions, whereas it is “deactivated” at the end of a session if a smaller capacity would be sufficient to clear all patients currently queueing.


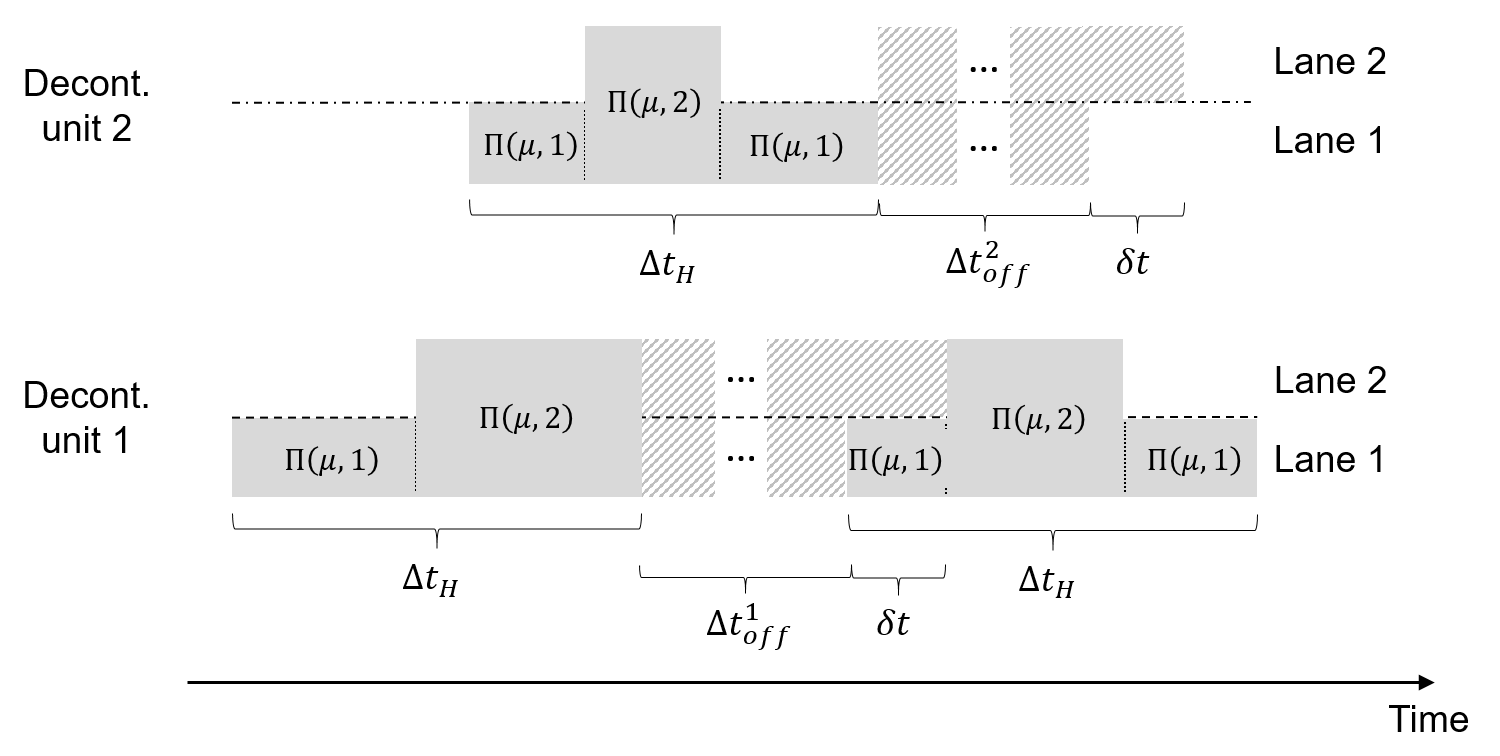


**Figure S2.** An illustration of decontamination process for emergency departments. Depending on the current number of patients queueing, lanes are activated with service rates depending on the number of active lanes in each decontamination unit. Once the session time ${\Delta t}_{H}$ has elapsed, the unit is used for team self-decontamination. The amount of time ${\Delta t}_{off}^{l}$ during which the whole decontamination unit is used for self-decontamination is computed as described in Appendix C. As soon as a lane is available the following team can start a new session.

Our algorithm (Algorithm S2) estimates the number of decontamination sessions needed by the generic emergency department $h$ (owning the set $L_{h}$ of decontamination units) to clear $n_{h}^{e}$ patients following event $e$ of type $\omega$. Decisions about system capacity are taken at regular time steps. The number of patients that can be treated with the current number of active lanes (“residual capacity”) is computed at each time step based on the current features of the system. The number of patients in queue is estimated using Stolletz’s method, approximating the $M(t)/M(t)/c(t)$ system with a particular (stationary) $M/M/c/c$ system at any given time interval, adapted to the case of heterogeneous servers (Saglam & Shahbazov, 2007).

Let us define:

- Time intervals $(t_{k-1},t_{k}], k=1,\ldots,T$, with $t_{T}=$ end of whole decontamination procedures
- $q_{k}=$ estimated queue length at the end of time interval $k:(t_{k-1},t_{k}]$
- $r_{k}=$ residual capacity $\equiv$ number of patients that would be decontaminated if system capacity at the end of time interval $\left( t_{k-1},t_{k} \right]$ is kept until the end of all ongoing sessions

Please note that there might be cases where decontamination is not required after a certain amount of time has elapsed since a HazMat event, corresponding, say, to people having changed clothes or showered prior to experiencing symptoms. In other words, for a given event involving self-presenters at emergency departments, decontamination procedures are assumed to cease at a given time instant $t_{T}$.

Our algorithm is based on the following rules:

- If $q_{k}=r_{k}$, then no action is taken (the current capacity is kept).
- If $q_{k}>r_{k}$ and at least one lane is inactive in the system, then a lane is chosen to be activated. The process is repeated until $q_{k}\leq r_{k}$, in which case the updated capacity of the system is able to treat all the patients currently waiting in queue.
- If $q_{k}<r_{k}$ and at least one lane is active in the system, then a lane is chosen to be deactivated. But if that lane is the only active one in its decontamination unit, then it is deactivated only if the corresponding decontamination session ends exactly at the current time point (note that we ensure that ${\Delta t}_{H}$ is a multiple of the length of the time frame $t_{k}-t_{k-1}$). The process is repeated (i.e. lanes are iteratively deactivated) ensuring that $q_{k}$ never exceeds $r_{k}$.

| **INITIALISATION** |
| --- |
| - $k:=1$ - $l\in L_{h}$ ordered (arbitrarily) by decreasing number of units ($w_{l}$) - Set $t_{0}=0$ (time of HazMat event) - Set $t_{T}$ (time at which the whole decontamination process ends) - Assumption: ${\Delta t}_{H}> {\Delta t}_{off}^{l}+\delta t$ - Set $t_{k}-t_{k-1}$ such that: $\frac{{\Delta t}_{H}}{t_{k}-t_{k-1}}\in\mathbb{N}^{+}$and $\frac{\delta t}{t_{k}-t_{k-1}}\in\mathbb{N}^{+}$ (i.e. each decontamination and self-decontamination session starts and ends exactly at the end of a time interval) - $t_{self}^{l}:=t_{0}-({\Delta t}_{off}^{l}+\delta t), l\in L_{h}$ (time when last self-decontamination has started in unit $l$ – initialised with an arbitrary negative value) - $t_{start}^{l}:=t_{0}-{\Delta t}_{H} , l\in L$ (decontamination session start time in unit $l$ – initialised by ensuring that $t_{k}=t_{start}^{l}+{\Delta t}_{H}, \forall l\in L_{h}$, at the first time step) - $s_{h}^{e}:=0$ (number of decontamination sessions required) |
| **ACTIVE LANES UPDATE** |
| $While k\leq T$  $For l=1 to \left\vert L_{h} \right\vert$  $If \left( t_{k}=t_{start}^{l}+{\Delta t}_{H} \right) then:$  $b_{l}:=0$ (number of active lanes in unit $l$)  $End If$  $End For$  **Compute** $\boldsymbol{q}_{\boldsymbol{k}}$ (cf. Appendix D)  **Compute** $\boldsymbol{r}_{\boldsymbol{k}}$ (cf. Appendix D)  $If (q_{k}>r_{k}) then$:  $While (q_{k}>r_{k}) and (\exists l: b_{l}<w_{l})$  $\bar{l}:=\min\left\{ l:b_{l}<w_{l} \right\}$  $b_{\bar{l}}:=b_{\bar{l}}+1$  $If b_{\bar{l}}=1 then:$  $t_{start}^{l}:=t_{k}$  $s_{h}^{e}:=s_{h}^{e}+1$  $End If$  **Compute** $\boldsymbol{r}_{\boldsymbol{k}}$  $End While$  $Else If (q_{k}<r_{k}) then:$  $While (q_{k}<r_{k} and \exists l: b_{l}>0)$  $If \left( \exists l: b_{l}=1 and t_{k}=t_{start}^{l}+{\Delta t}_{H} \right) then:$  $\bar{l}:=\max\left\{ l: b_{l}=1 and t_{k}=t_{start}^{l}+{\Delta t}_{H} \right\}$  $b_{\bar{l}}:=b_{\bar{l}}-1$  $t_{self}^{l}:=t_{k}$  $Else If \left( \exists l: b_{l}>1 \right):$  $\bar{l}:=\max\left\{ l: b_{l}>1 \right\}$  $b_{\bar{l}}:=b_{\bar{l}}-1$  $End If$  **Compute** $\boldsymbol{r}_{\boldsymbol{k}}$  $If (q_{k}>r_{k}) then:$  $b_{\bar{l}}:=b_{\bar{l}}+1$  $If \left( b_{\bar{l}}=1 \right) then:$  $t_{self}^{\bar{l}}:=t_{k}-({\Delta t}_{off}^{\bar{l}}+\delta t)$  $End If$  EXIT *While*  $End If$  $End While$  $End If$  $k:=k+1$ |

**Algorithm S2.** Algorithm to compute the number of decontamination sessions required by emergency department $h$ in response to event $e$.

**4. Allocation strategy**

Based on our “plausible worst-case scenario” approach, the global protective suit demand for each healthcare service responsible for an LRF corresponds to the maximum single-event protective suit demand estimate across all events with likelihood level equal to or above a given threshold in that region.

Allocation strategy for emergency departments

Given a likelihood level threshold $\bar{p}$, the protective suit demand estimate $d_{h}\left( \bar{p} \right)$ for emergency department $h$ in LRF $r$ is chosen to allow resilience to any single event of likelihood level equal to or above $\bar{p}$ in LRF $r$:

|  | $d_{h}\left( \bar{p} \right)=max\left\{ d_{h}^{e}, e\in E : p_{r}^{e}\geq\bar{p} \right\}$. | (8) |
| --- | --- | --- |

Allocation strategy for ambulance services

Given a likelihood level threshold $\bar{p}$, the protective suit demand estimate $d_{a}\left( \bar{p} \right)$ for ambulance service $a$ is chosen to allow resilience to any single event of likelihood level equal to or above $\bar{p}$ in all relevant LRFs:

|  | $d_{a}\left( \bar{p} \right)=max\left\{ d_{a}^{e}, e\in E :\exists r\in R_{a} with p_{r}^{e}\geq\bar{p} \right\}$. | (9) |
| --- | --- | --- |

**Appendix A**

We assume a service rate decrease when more than one lane are active (the active lanes of a decontamination unit share the same team). We model this using an exponential decay function and assuming that when two lanes are used then the service rate of each lane will be 20% lower than $\mu$. The function used is the following:

|  | $\pi\left( \mu,b_{l} \right)=\mu\cdot e^{-\sigma(b_{l}-1)}$. | (A.1) |
| --- | --- | --- |

We can observe that $\pi\left( \mu,1 \right)=\mu$. We only need to identify the parameter $\sigma$ such that $\pi\left( \mu,2 \right)=0.8\cdot\mu$. We do that by imposing $e^{-\sigma(b_{l}-1)}=0.8$ that gives us $\sigma=-\ln0.8$.

Therefore, the service rate for each lane (i.e. the average service rate) in a generic decontamination unit if $b_{l}$ lanes are active is given by the function:

|  | $\pi\left( \mu,b_{l} \right)=\mu\cdot e^{(\ln0.8)\cdot(b_{l}-1)}$. | (A.2) |
| --- | --- | --- |

Thus, the total service rate in decontamination unit $l$ if $b_{l}$ lanes are active is:

|  | $\Pi\left( \mu,b_{l} \right)=b_{l}\cdot\pi\left( \mu,b_{l} \right)=b_{l}\cdot\mu\cdot e^{(\ln0.8)\cdot(b_{l}-1)}$. | (A.3) |
| --- | --- | --- |

Total service rate of the system when $b_{l}$ lanes are active in a decontamination unit $l$:

|  | $\Pi_{tot}(\mu,b_{1}, \ldots, b_{L})=\sum_{l=1}^{L} \Pi\left( \mu,b_{l} \right)$. | (A.4) |
| --- | --- | --- |

**Appendix B**

Let us define: $\vartheta_{r}=$ size $\left( \frac{km^{2}}{{10}^{3}} \right)$ of LRF $r$ and please recall that $\left| H_{r} \right|$ is the number of EDs in LRF $r$. The density of EDs in LRF $r$ is then given by: $\delta_{r}=\frac{\left| H_{r} \right|}{\vartheta_{r}}$.

Using a generalised version of the logistic function, the proportion of casualties to be assigned to each ED in LRF $r$ is given by (Figure S3):

|  | $\alpha_{r}\left( \delta_{r} \right)=\left\{ \begin{aligned} 1 & , if \left\vert H_{r} \right\vert=1 \\ y_{2}-\frac{y_{2}-y_{1}}{1+e^{-\left( \delta_{r}-x \right)}}, if \left\vert H_{r} \right\vert>1 \end{aligned} \right.$ | (B.1) |
| --- | --- | --- |

Parameters:

- $y_{1}\in[0,1)$ is the lower horizontal asymptote of $\alpha_{r}$ (i.e. the smallest acceptable proportion of casualties);
- $y_{2}=1$ is the upper horizontal asymptote of $\alpha_{r}$ (i.e. the biggest acceptable proportion of casualties);
- $x=3$ determines the intercept of $\alpha_{r}$ with the vertical axis. It is arbitrarily chosen as the smallest integer allowing a proportion of casualties of at least 0.9 in LRFs with less than 1 ED per $\frac{km^{2}}{{10}^{3}}$.


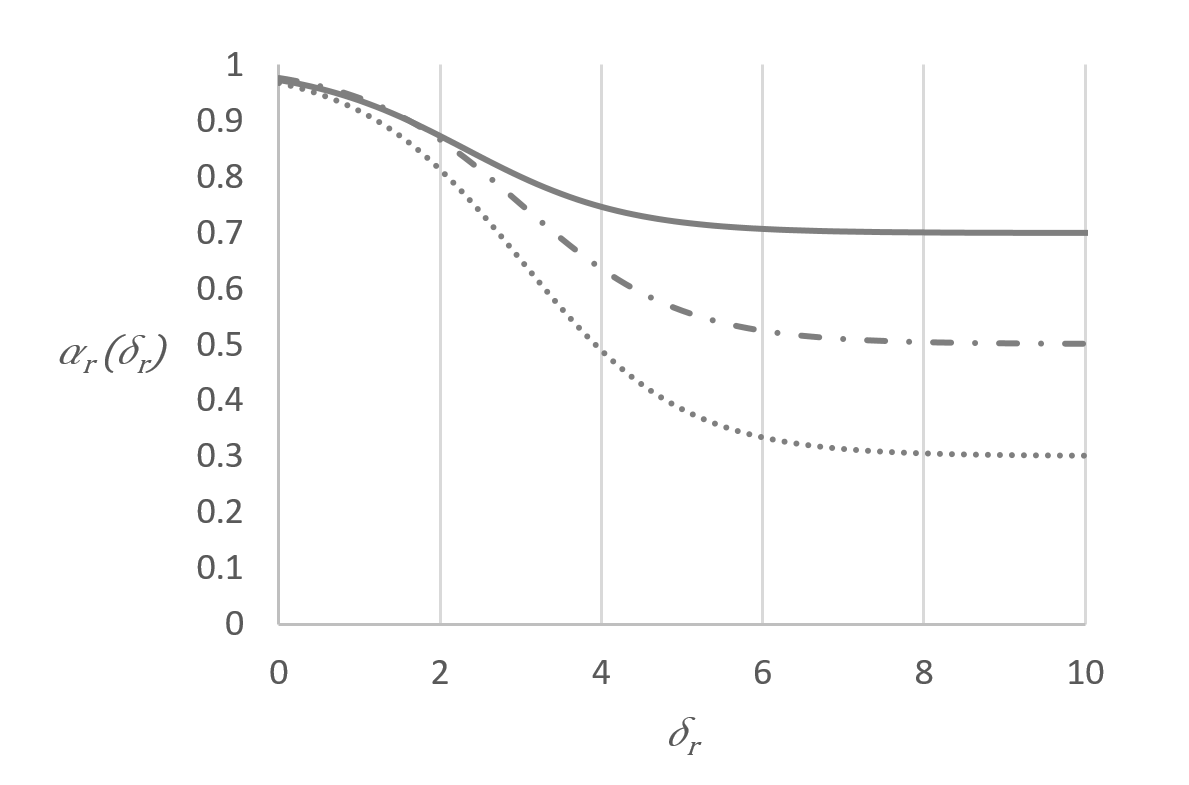


**Figure S7.** Plot of $\alpha_{r}\left( \delta_{r} \right)$ for $\left| H_{r} \right|>1$, for different levels of $y_{1}$. The proportion of $n_{h}^{e}$ assigned to each emergency department depends on the density $\delta_{r}$ of EDs in LRF $r$.

**Appendix C**

The expression $\left\lceil\frac{m_{j}}{\eta} \right\rceil$ (with $j\in\left\{ "A","H" \right\}$) represents the number of self-decontamination sessions (each occupying a lane for $\delta t$ time units) that are required at the end of each decontamination session in each unit.

We also define the number of self-decontamination sessions $o_{l}$ that are carried out while the following team is already operating (i.e. the $\delta t$ slots indicated in Figure and Figure S2), as these will be used for computing the total service rate of the system:

|  | $o_{l}:=\left\lceil\frac{m_{j}}{\eta} \right\rceil mod w_{l}$ | (C.1) |
| --- | --- | --- |

By construction, the amount of time a decontamination unit $l$ is completely busy for self-decontamination is given by the following formula:

|  | ${\Delta t}_{off}^{l}=\frac{\left\lceil\frac{m_{j}}{\eta} \right\rceil-o_{l}}{w_{l}}\cdot\delta t$ | (C.2) |
| --- | --- | --- |

Please note that the quantity ${\Delta t}_{off}^{l}$ is not explicitly computed for ambulance services in this work.

**Appendix D**

**Sub-routines for emergency departments**

We report here the two sub-routines for computing $q_{k}$ and $r_{k}$ at each time step of the algorithm described in Algorithm S2.

**Compute** $\boldsymbol{r}_{\boldsymbol{k}}$

$r_{k}$ is the number of patients that could be potentially treated if system capacity at the end of time interval $\left( t_{k-1},t_{k} \right]$ is kept until the end of all current decontamination sessions.

At time $t_{k}$, the number of patients $r_{k}^{l}$ that could be treated in decontamination unit $l$ until the end of the current session can be greater than zero only if:

|  | $t_{start}^{l}\leq t_{k}\boldsymbol{<}t_{start}^{l}+{\Delta t}_{H}$ | (D.1) |
| --- | --- | --- |

By construction (cf. Algorithm S2), we also have:

|  | $r_{k}^{l}>0\Rightarrow t_{self}^{l}\leq t_{start}^{l}$ | (D.2) |
| --- | --- | --- |

Under validity of the conditions above, and considering our assumption ${\Delta t}_{H}> {\Delta t}_{off}^{l}+\delta t$, we can distinguish the following three cases to compute $r_{k}^{l}$:

| **Case A:** $t_{self}^{l}\leq t_{k}\leq t_{self}^{l}+{\Delta t}_{off}^{l}$  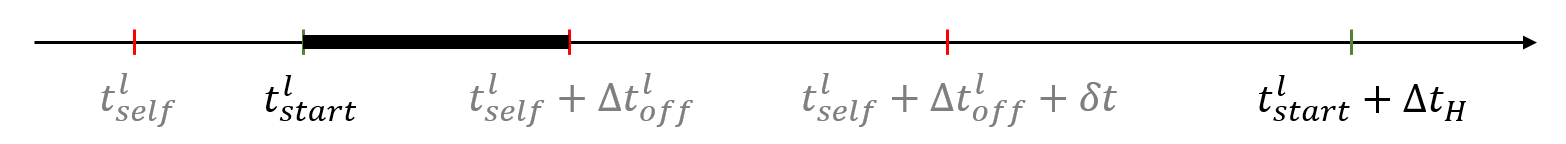  $\Rightarrow r_{k}^{l}=\Pi\left( \mu,w_{l}-o_{l} \right)\cdot\delta t+$  $+\Pi\left( \mu,w_{l} \right)\cdot[t_{start}^{l}+{\Delta t}_{H}-\left( t_{self}^{l}+{\Delta t}_{off}^{l}+\delta t \right)]$ |
| --- |
| **Case B:** $t_{self}^{l}+{\Delta t}_{off}^{l}<t_{k}\leq t_{self}^{l}+{\Delta t}_{off}^{l}+\delta t$  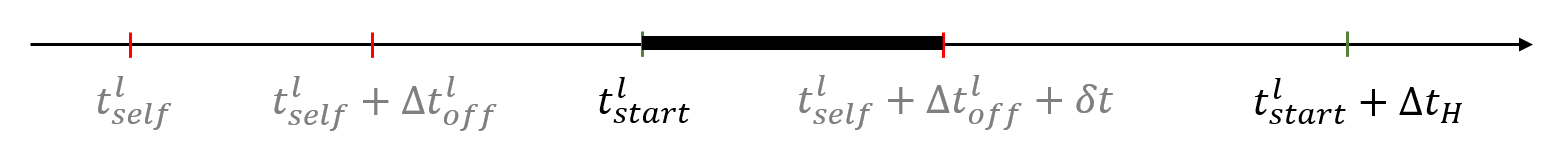  $\Rightarrow r_{k}^{l}=\Pi\left( \mu,w_{l}-o_{l} \right)\cdot\left[ t_{self}^{l}+{\Delta t}_{off}^{l}+\delta t-t_{k} \right]+$  $+\Pi\left( \mu,w_{l} \right)\cdot[t_{start}^{l}+{\Delta t}_{H}-\left( t_{self}^{l}+{\Delta t}_{off}^{l}+\delta t \right)]$ |
| **Case C:** $t_{self}^{l}+{\Delta t}_{off}^{l}+\delta t<t_{k}$  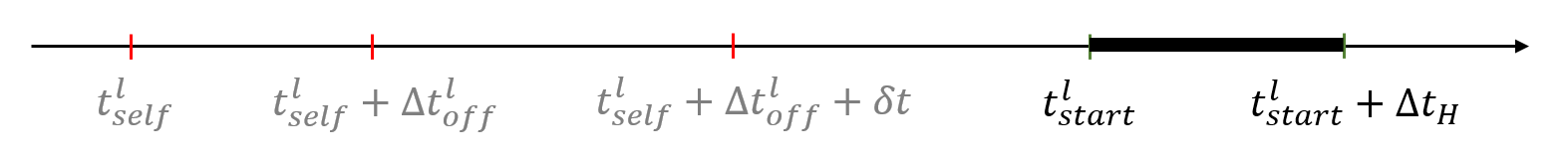  $\Rightarrow r_{k}^{l}=\Pi\left( \mu,w_{l} \right)\cdot[t_{start}^{l}+{\Delta t}_{H}-t_{k}]$ |

**Algorithm S3.** Formulas for computing $r_{k}^{l}$ based on when the current decontamination session has started relative to when the last team self-decontamination session ended.

$r_{k}$ is then given by:

|  | $r_{k}=\sum_{l\in L_{h}} r_{k}^{l}$ | (D.3) |
| --- | --- | --- |

**Compute** $\boldsymbol{q}_{\boldsymbol{k}}$

Queue length at the end of time interval $\left( t_{k-1},t_{k} \right]$ is computed using an approximation method by Stolletz (2008). An estimation of $q_{k}$ is determined after approximating the original $M(t)/M(t)/c(t)$ system with a stationary $M_{k}/M_{k}/c_{k}/c_{k}$ system with the following features:

- average arrival rate during period $k$:

|  | $\lambda_{k}=\frac{1}{t_{k}-t_{k-1}} \int_{t_{k-1}}^{t_{k}} \lambda\left( t \right)dt, k=1,\ldots, T$ | (D.4) |
| --- | --- | --- |

- average service rate (of each server) during period $k$:

|  | $\mu_{k}=\frac{1}{t_{k}-t_{k-1}} \int_{t_{k-1}}^{t_{k}} \mu\left( t \right)dt, k=1,\ldots, T$ | (D.5) |
| --- | --- | --- |

From the $M_{k}/M_{k}/c_{k}/c_{k}$ system, the non-served customers (patients) in time interval $\left( t_{k-1},t_{k} \right]$ are carried over into time interval $\left( t_{k},t_{k+1} \right]$. The number of blocked customers is estimated using the Erlang-loss formula (Allen, 1990):

|  | $\beta_{k}=\tilde{\lambda}_{k}\cdot P_{k}\left( B \right)=\tilde{\lambda}_{k}\cdot\frac{\left( {\tilde{\lambda}_{k}}/{\mu_{k}} \right)^{c_{k}}}{c_{k}! \sum_{j=0}^{c_{k}} \frac{\left( {\tilde{\lambda}_{k}}/{\mu_{k}} \right)^{j}}{j!}}$ | (D.6) |
| --- | --- | --- |

where $\tilde{\lambda}_{k}$ is an artificial arrival rate accounting for blocked customers in the previous time interval and $P_{k}\left( B \right)$ is the steady state probability of blocking during the current time interval.

The artificial arrival rate is computed recursively to account for these customers:

|  | $\tilde{\lambda}_{k}=\lambda_{k}+\beta_{k-1}=\lambda_{k}+\tilde{\lambda}_{k-1}\cdot P_{k-1}(B)$ | (D.7) |
| --- | --- | --- |

The expected number of waiting customers (queue length) at the end of time interval $\left( t_{k},t_{k+1} \right]$ is determined by multiplying the estimated number of blocked customers by the interval length.

|  | $q_{k+1}=\beta_{k+1}\cdot(t_{k+1}-t_{k})$ | (D.8) |
| --- | --- | --- |

However, the formula for $P_{k}\left( B \right)$ reported above cannot be directly used in our study due to the particular features of the system. Indeed, this formula uses the quantities $\left( {\tilde{\lambda}_{k}}/{\mu_{k}} \right)^{c_{k}}$ and $\left( {\tilde{\lambda}_{k}}/{\mu_{k}} \right)^{j}$ implying the same service rate $\mu_{k}$ for all $c_{k}$ servers, but our system is formed by servers that might have different service rates at some time points.

Saglam & Shahbazov (2007) proposed a generalisation of the Erlang-loss formula to the case of heterogeneous servers.

Let us consider the single offered loads of each servers in our $M_{k}/M_{k}/c_{k}/c_{k}$ system:

|  | $\frac{\tilde{\lambda}_{1}}{\mu_{1}}, \frac{\tilde{\lambda}_{2}}{\mu_{2}}, \frac{\tilde{\lambda}_{3}}{\mu_{3}},\ldots,\frac{\tilde{\lambda}_{c_{k}}}{\mu_{c_{k}}}$ | (D.9) |
| --- | --- | --- |

The k-th elementary symmetric functions of the above listed offered loads can be defined as follows:

|  | $E_{0}=1$ | (D.10) |
| --- | --- | --- |
|  | $E_{j}=\sum_{1\leq\xi_{1}<...<\xi_{j}\leq c_{k}} \left( \frac{\tilde{\lambda}_{\xi_{1}}}{\mu_{\xi_{1}}} \right)\cdot\left( \frac{\tilde{\lambda}_{\xi_{2}}}{\mu_{\xi_{2}}} \right)\cdot\left( \frac{\tilde{\lambda}_{\xi_{3}}}{\mu_{\xi_{3}}} \right)\cdot\cdot\cdot\left( \frac{\tilde{\lambda}_{\xi_{k}}}{\mu_{\xi_{k}}} \right), 0\leq j\leq c_{k}$ | (D.11) |

where the summation above extends over all $Z\left( c_{k},j \right)=\left( \begin{matrix} c_{k} \\ j \end{matrix} \right)$ combinations of $j$ distinct elements $\{\xi_{1},\ldots,\xi_{k}\}$ from the set $\{1,\ldots,c_{k}\}$.

The quantity $W_{j}=\frac{E_{j}}{Z\left( c_{k},j \right)}$ can then be used to generalise $\left( {\tilde{\lambda}_{k}}/{\mu_{k}} \right)^{j}$, leading to the following expression for $P_{k}\left( B \right)$ which we used in our algorithm:

|  | $P_{k}\left( B \right)=\frac{W_{c_{k}}}{c_{k}! \sum_{j=0}^{c_{k}} \frac{W_{j}}{j!}}$ | (D.12) |
| --- | --- | --- |

**References**

Kilic, A., Dincer, M. C., & Gokce, M. A. (2014). Determining optimal treatment rate after a disaster. Journal of the Operational Research Society, 65(7), 1053–1067. http://doi.org/10.1057/jors.2013.52

Stolletz, R. (2008). Approximation of the non-stationary M(t)/M(t)/c(t)-queue using stationary queueing models: The stationary backlog-carryover approach. European Journal of Operational Research, 190(2), 478–493. http://doi.org/10.1016/j.ejor.2007.06.036

Saglam, V., & Shahbazov, A. (2007). Minimizing loss probability in queuing systems with heterogeneous servers. Iranian Journal of Science and Technology, 31(2), 199–206.

Allen, A. O. (1990). Probability, statistics, and queueing theory with computer science applications. Boston: Academic Press : Harcourt Brace Jovanovich. Retrieved from http://public.eblib.com/choice/publicfullrecord.aspx?p=1901176
